# Editorial: Celebrating the year of Ramon y Cajal: cellular biology of the retina

**DOI:** 10.3389/fncel.2025.1754379

**Published:** 2026-01-06

**Authors:** Nicolás Cuenca, M. Valeria Canto-Soler, S. Patricia Becerra

**Affiliations:** 1 Department of Physiology, Genetics and Microbiology, University of Alicante, Alicante, Spain; 2 CellSight Ocular Stem Cell and Regeneration Research Program, Department of Ophthalmology, Sue Anschutz-Rodgers Eye Center, University of Colorado, Aurora, CO, United States; 3 Section of Protein Structure and Function, National Eye Institute, National Institutes of Health, Bethesda, MD, United States

**Keywords:** eye development, ocular biology, retina physiology, retina therapeutic strategies, retinal cell biology, retinal degenerative diseases, retinal development

The figure of Santiago Ramón y Cajal (1852–1934) and his groundbreaking work on the organization of the nervous system and the Neuron Theory are widely recognized. His monumental contributions rightly earned him the title of the founder of modern neuroscience. Alongside Camillo Golgi, Cajal was awarded the Nobel Prize in Physiology or Medicine in 1906 for their pioneering studies on the structure of the nervous system. While Golgi adhered to the reticular theory, Cajal used Golgi's staining method to reach a profoundly different conclusion, one that culminated in the neuron doctrine, which became a cornerstone of neuroscience.

Despite his extensive contributions, Cajal's studies on the structure and neuronal types of the retina remain relatively overlooked. His seminal work, “*La rétine des vertébrés*” wrote in French (Ramón y Cajal, 1892), translated in English (Ramón y Cajal, 1972) and Spanish (Ramón y Cajal, 2021), is among his most remarkable achievements. Cajal recognized the retina as an ideal model for uncovering the characteristics of nerve cells that could be generalized to the entire central nervous system. His meticulous investigations into the structural organization of the retina, combined with his visionary predictions about its functions, laid the foundation for the neuroanatomy, neurophysiology, and neuropathology of this critical sensory organ. The general organizational framework of the retina proposed by Cajal remains fundamentally valid, with many aspects of his work continuing to hold relevance today. His detailed descriptions of retinal cell types and their intricate interconnections, observed across various species, rank among the most comprehensive studies ever conducted. In his memoirs, Cajal expressed deep fondness for studying the retina, calling it the “oldest and most steadfast” of his scientific passions.

Cajal's contributions to retinal research have shaped our understanding of the nervous system, particularly the retina. He meticulously described and classified various retinal cells, including photoreceptors, bipolar cells, amacrine cells, and ganglion cells, across multiple vertebrate species. His studies of the sublaminae of the inner plexiform layer revealed the organizational complexity of retinal nerve cells and their roles in visual signal processing. His discovery of centrifugal fibers connecting to amacrine cells underscored the retina's intricate network and interconnectivity. Cajal also identified cellular convergence circuits essential for visual information processing and described interplexiform cells, which link different retinal layers to facilitate efficient visual integration.

Cajal's work extended to Müller cells, which he demonstrated to provide structural support, protection, and ionic balance within the retina. Additionally, he offered critical insights into retinal histogenesis during embryonic development, elucidating how retinal cells form and organize. His detailed description of the specialized structure of the fovea deepened our understanding of high-resolution vision. Finally, Cajal proposed a chemotactic theory of neural connection formation, suggesting that chemical signals guide nerve cells during development.

Cajal's pioneering research on the retina remains a cornerstone of retinal studies and continues to influence broader neuroanatomical and neurophysiological research. His legacy as a visionary scientist endures, with his insights remaining as relevant and inspiring as ever in the field of neuroscience.

This Research Topic pays tribute to Santiago Ramón y Cajal in celebration of the 170th anniversary of his birth by collecting recent advances in the study of retinal cellular biology and methods applied to better understand this complex system. This Research Topic includes eight original research articles, one brief research report, one perspective, and four review articles. These articles encompass various aspects of ocular biology, including molecular pathways that regulate eye and retina development, mechanisms involved in retinal disease, and potential therapeutic strategies. Importantly, these studies are conducted using a range of relevant systems, including rodents, zebrafish, and human samples, as well as emerging models such as human retinal organoids and the Mongolian gerbil.

Retinal neurodegenerative diseases such as age-related macular degeneration, glaucoma, diabetic retinopathy, and retinitis pigmentosa have distinct etiologies but share common cellular and molecular responses to injury, including inflammation, oxidative stress, and apoptosis (Cuenca et al., 2014). This Research Topic includes three review articles that focus on the mechanisms underlying these conditions.

Glaucoma is a multifactorial disease characterized by the degeneration of retinal ganglion cells and axonal loss. In this context, Fernández-Albarral et al. review the molecular mechanisms that contribute to its development, including mitochondrial dysfunction, oxidative stress, vascular dysregulation, and neuroinflammation, all factors that may be exacerbated by aging. The authors also address the central role of glial cells (microglia, astrocytes, and Müller cells), whose activation may initially be protective, but when sustained, contributes to neurodegeneration by inducing a pro-inflammatory state, as well as the influence of genetic factors and microRNAs, suggesting that modulating the immune response and glial activation are promising approaches for treating the disease.

The study from Bighinati et al. reviews the molecular mechanisms underlying hereditary photoreceptor degeneration, specifically in the context of retinitis pigmentosa. It explores various cell death pathways, such as oxidative stress, inflammation, elevated cGMP levels, calcium imbalance, and endoplasmic reticulum stress caused by misfolded proteins like mutant rhodopsin. The authors detail how each of these factors contributes to disease progression and, crucially, discuss both preclinical and clinical therapeutic approaches targeting these common pathways to preserve visual function and photoreceptor survival, with a focus on gene-agnostic strategies.

Syntaxins are a family of proteins essential for membrane fusion events, specifically within the retina. While syntaxins are known to play key roles in neurotransmitter release and protein trafficking, their specific functions in the retina have been less well-studied. Tebbe et al. present a thorough review of the distribution and role of different syntaxins, including STX1, STX2, STX3, and STX4, within retinal layers, their involvement in synaptic transmission in photoreceptor and horizontal cells, and in the trafficking of critical proteins. The study also highlights an emerging association between mutations in syntaxins, particularly STX3, and retinal diseases such as early-onset severe retinal dystrophy, underscoring the importance of these proteins for photoreceptor cell health and survival.

In addition to these review articles, two original research studies further address mechanisms involved in retinal degenerative conditions.

Diabetic retinopathy is a metabolic disorder characterized by molecular and cellular alterations that affect both vascular and neuronal components of the retina, ultimately leading to blindness (Albertos-Arranz et al., 2025). The study by Nam et al. reveals the therapeutic potential of peptain-1 in preventing retinal damage, with a focus on capillary degeneration and neuroinflammation induced by ischemia/reperfusion (I/R) in mice. Their study demonstrates that peptain-1 can block apoptosis of human retinal endothelial cells, a key pathological feature of diabetic retinopathy. Moreover, administration of peptain-1 protects retinal capillaries from I/R-induced damage and attenuates microglial activation and pro-inflammatory cytokine levels in the retina, supporting its potential as a novel treatment for the early stages of ischemic retinal diseases.

Taskintuna et al. address another emerging factor influencing retinal degeneration by exploring how sex-specific differences affect retina and the retinal pigment epithelium function, especially in the context of aging and suppression of the *Pgc-1*α gene in mice. Their findings show that *Pgc-1*α inhibition leads to a sex-dependent decline in RPE and retinal function, with females exhibiting greater susceptibility to age-related deterioration. The study further reveals that *Pgc-1*α differentially regulates genes involved in antioxidant defense and mitochondrial dynamics in males and females, suggesting that distinct mechanisms of oxidative stress management and mitochondrial function may underlie these disparities. These insights offer promising directions for the development of sex-specific treatments for age-related macular degeneration.

This Research Topic also brings together five studies that illuminate key mechanisms guiding retinal development and their disruption in disease.

Hofstetter et al. reveal that the Rho GTPase *Cdc42* is essential for optic cup morphogenesis in mice. Loss of *Cdc42* disrupts polarity, cytoskeletal organization, and progenitor proliferation, leading to microphthalmia and coloboma, highlighting its critical role in early eye formation.

Kar et al. use serial electron microscopy to uncover a novel population of “inner” Müller glia in the fovea of a human born preterm. These glial cells, structurally distinct from classical Müller cells, may reflect adaptations to disrupted neuronal migration during development.

Dalmaso et al. show that deletion of the platelet-activating factor receptor (PAFR) alters post-natal retinal development in mice, increasing progenitor proliferation while impairing neuronal differentiation and synaptic transmission. Their findings identify PAFR as a key regulator of neurogenic balance and circuit maturation.

The study by Farre et al. leverages the similarities between the zebrafish retina and the human retina to deepen our understanding of transcriptional heterogeneity in cones that express tandemly replicated opsins and the mechanisms regulating such differential expression. Results from this study suggest that multiple genes within the cones expressing tandemly replicated opsins are non-stochastically regulated, and that multiple signals likely control the transcriptional differences between cone subtypes. These findings contribute to a deeper understanding of the mechanisms that regulate the development of retinal cone subtypes.

Finally, Zheng and Chen review 25 years of research on the transcription factor *CRX*, central to photoreceptor gene regulation. They highlight distinct disease mechanisms linked to *CRX* variants and discuss emerging systems biology tools that promise to inform precision therapies.

Together, these contributions underscore the importance of cytoskeletal dynamics, glial specialization, lipid signaling, and transcriptional control in shaping retinal architecture and function.

Current animal models continue to provide invaluable insights into the field of retinal biology; however, their transferability to human retinal biology presents significant limitations due to differences between the human retina and these models. Thus, the development and validation of new human-relevant models to study the mechanisms regulating retinal function in both normal and diseased conditions remain essential.

Contributing to this important effort, Günter et al. report the characterization of a diurnal rodent, the Mongolian gerbil (*Meriones unguiculatus*) (MG), which features a cone-rich retina and a macula-like specialized region. The study focuses on evaluating the MG's cone system functionality using full-field electroretinography (ERG), together with a morphological assessment of the retina and macula-like region via angiography, optical coherence tomography (OCT), and immunohistochemistry. The authors conclude that the MG retina exhibits features similar to those observed in the human retina, thereby providing a suitable model for investigating cone system physiology, pathophysiology, and potentially testing novel therapeutic strategies.

Importantly, human retinal organoids are emerging as powerful, human-relevant models that can recreate, to a certain extent, the cellular composition and organization of the native retina, while providing precise temporal resolution and control over environmental conditions (Vergara et al., 2024). An important consideration, however, is the inherent variability of this *in vitro* system and its implications for research outcomes and their interpretation.

Within this context, Carido et al. assessed the reproducibility, efficiency, and variance of a previously published protocol for generating human retinal organoids, known as the CYST protocol (Zhu et al., 2013; Lowe et al., 2016; Volkner et al., 2022). The study evaluated organoid development, efficiency, and reproducibility, including major retinal cell type composition and its variance across seven hiPSC lines derived by different methods and from various cell sources. Furthermore, the study proposed a potential strategy for systematically evaluating different organoid protocols in a side-by-side comparison. Another important consideration for using retinal organoids for disease modeling is the ability of the *in vitro* system to recapitulate disease hallmarks observed in human patients.

The study conducted by James et al. reports the development and characterization of a novel human retinal organoid (RO) model derived from induced pluripotent stem cells (iPSCs) from patients with familial Alzheimer's disease (AD). Notably, AD pathology is found not only in the brain but also in the retinas of AD patients, hence, retinal features of AD are being evaluated as potential biomarkers for disease diagnosis and the evaluation of disease progression. Importantly, this study demonstrated that AD-retinal organoids recapitulate the primary histopathological hallmarks of AD, offering a novel tool for drug screening, biomarker discovery, and pathophysiological studies.

Finally, in a Perspective article, Tsukamoto presents an interesting example of the potential synergy between neuroscience and connectome via artificial intelligence. Specifically, the author discusses the application of convolutional neural network (CNN), a machine learning algorithm robust for classifying neuron borderlines on electron micrograph images for automated connectomic analysis, to study the primary rod signal pathway in mouse and macaque retinas with special reference to electrical synapses.

Altogether, this Research Topic reflects the substantial progress made in retinal research since Cajal's pioneering work. The studies presented here advance our understanding of retinal development, cellular physiology, and degenerative mechanisms, while underscoring the growing importance of human-relevant models. In doing so, they not only honor Cajal's foundational contributions but also highlight emerging directions that will inform future efforts to uncover disease mechanisms and develop effective therapies to preserve vision.
